# A porcine model of postoperative hemi-diaphragmatic paresis to evaluate a unilateral diaphragmatic pacemaker

**DOI:** 10.1038/s41598-023-39468-w

**Published:** 2023-08-03

**Authors:** Tobias Kratz, Roman Ruff, Marit Bernhardt, David Katzer, Ulrike Herberg, Boulos Asfour, Johannes Breuer, Christina Oetzmann von Sochaczewski, Benjamin Bierbach

**Affiliations:** 1 Department of Paediatric Cardiology, University Hospital Bonn, Bonn, Germany; 2 Fraunhofer IBMT, Institute for Biomedical Engineering, Sulzbach, Germany; 3 Institute of Pathology, University Hospital Bonn, Bonn, Germany; 4 Department of Paediatrics, University Hospital Bonn, Bonn, Germany; 5 Department of Paediatric Cardiology, University Hospital Aachen, Aachen, Germany; 6 Department of Paediatric Cardiac Surgery, University Hospital Bonn, Bonn, Germany; 7 Department of Surgery, Division of Paediatric Surgery, University Hospital Bonn, Bonn, Germany

**Keywords:** Experimental models of disease, Paediatric research

## Abstract

Unilateral phrenic nerve damage is a dreaded complication in congenital heart surgery. It has deleterious effects in neonates and children with uni-ventricular circulation. Diaphragmatic palsy, caused by phrenic nerve damage, impairs respiratory function, especially in new-borns, because their respiration depends on diaphragmatic contractions. Furthermore, Fontan patients with passive pulmonary perfusion are seriously affected by phrenic nerve injury, because diaphragmatic contraction augments pulmonary blood flow. Diaphragmatic plication is currently employed to ameliorate the negative effects of diaphragmatic palsy on pulmonary perfusion and respiratory mechanics. This procedure attenuates pulmonary compression by the abdominal contents. However, there is no contraction of the plicated diaphragm and consequently no contribution to the pulmonary blood flow. Hence, we developed a porcine model of unilateral diaphragmatic palsy in order to evaluate a diaphragmatic pacemaker. Our illustrated step-by-step description of the model generation enables others to replicate and use our model for future studies. Thereby, it might contribute to investigation and advancement of potential improvements for these patients.

## Introduction

Unilateral phrenic nerve palsy and subsequent hemi-diaphragmatic paresis has been^1^ and still is^2^ not an uncommon complication of paediatric cardiac surgery. Unilateral hemi-diaphragmatic paralysis results in increased mortality, morbidity, tracheostomy rates, and prolonged mechanical ventilation^2^. While some favoured a conservative approach^3^, others opted for early diaphragmatic plication^4^. However, comparative evaluation of both approaches showed similar success rates: 60% of affected patients improved^5^. *Kaufman* and co-workers also performed phrenic nerve reconstruction by neurolysis and sural nerve grafting in adults^6^, but this approach remained scattered and size constraints preclude transferral to the paediatric patient. Phrenic nerve palsy is of particular relevance in two subpopulations in congenital cardiac surgery.

The first group consists of children with Fontan circulation: In these patients, pulmonary blood flow is established without a supporting ventricle. The elevated central venous pressure actuates pulmonary blood flow, assisted by the diaphragm’s suction effect during inspiration. Therefore, pulmonary blood flow depends on diaphragmatic function in Fontan patients, which are severely affected from diaphragmatic palsy^7^. Neonates undergoing heart surgery represent the second group and suffer more from diaphragmatic palsy compared to older children^8^, because the neonate’s respiratory mechanic depends largely on the diaphragmatic function^9^.

Currently, the available systems for diaphragmatic pacing have been developed for bilateral use, for example following high cervical trauma^10^, congenital central hypoventilation syndrome^11^, and amyotrophic lateral sclerosis^12^. In the clinical setting of unilateral phrenic nerve injury, the challenge remains the stimulation of the affected hemi-diaphragm. A pacing system, in the sense of a closed loop, is non-existent at present for this situation. In order to facilitate preclinical testing of a totally implantable and triggered unilateral diaphragmatic pacemaker, we aimed to develop a pre-clinical large animal model of postoperative hemi-diaphragmatic paresis.

## Results

**Figure 1:**
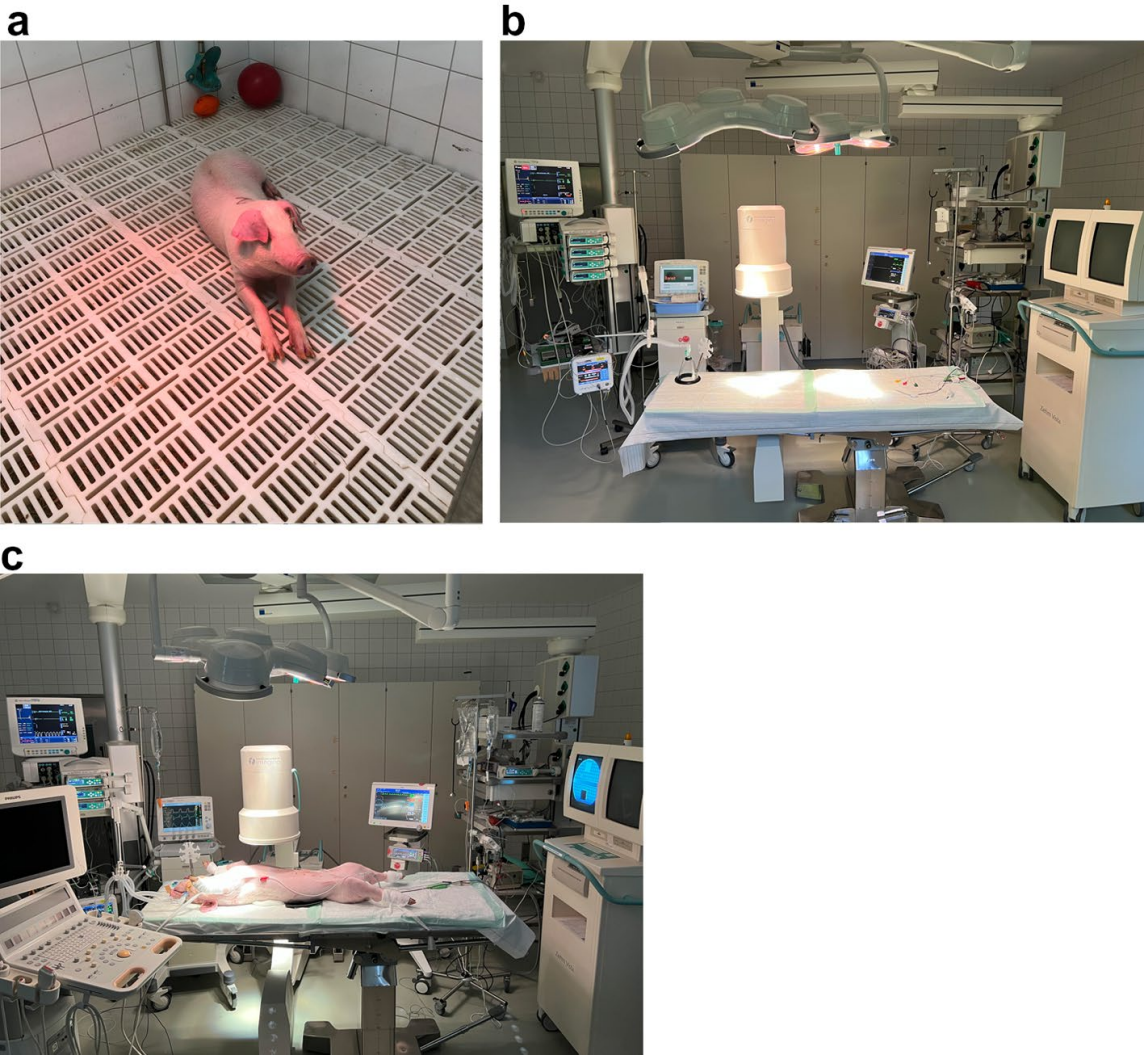
Swine and the experimental setup. (**a**) A representative swine in its box laying partially under the heat lamp with some of the material to play with in the background. (**b**) Our operation room with its full setup without the swine and without the echo. (**c**) The full setup for our experiment before preparation and application of the surgical drapes to the pig.

**Figure 2:**
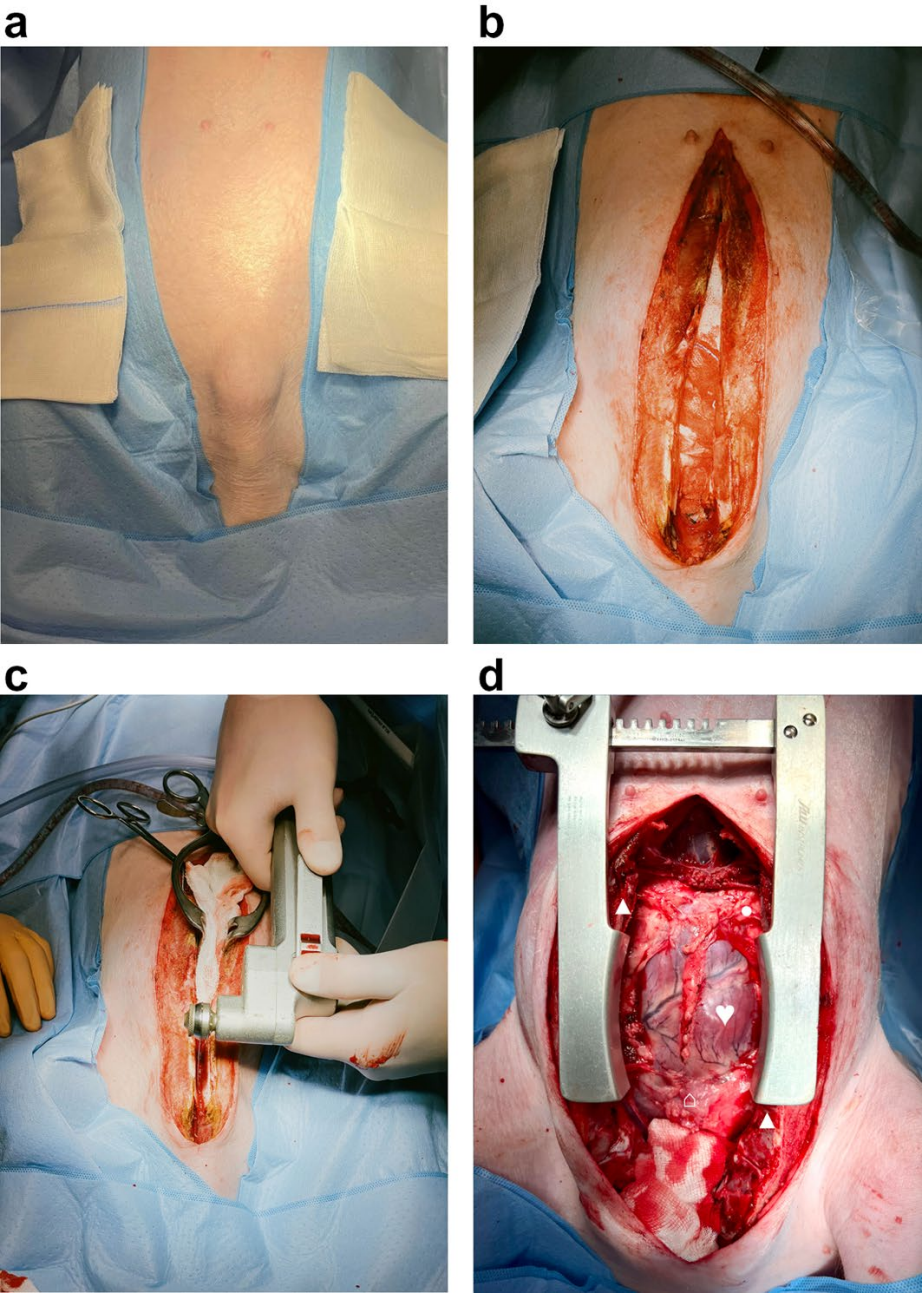
Getting access to the heart. The orientation is the same in all pictures: the rostral direction is at the bottom and the caudal direction at the top of the picture. (**a**) Pre-operative situation after full draping of the swine. (**b**) Skin incision via blade and monopolar preparation of the subcutaneous tissues until the sternum was exposed. (**c**) Median sternotomy using an oscillating bone saw. (**d**) Full exposure of the heart from its base to the apex and bilateral access to both hemi-diaphragms. ♥ designates the heart.▲ designates the sternum. ● designate the pericardium. ⌂ designates the thymus.

**Figure 3:**
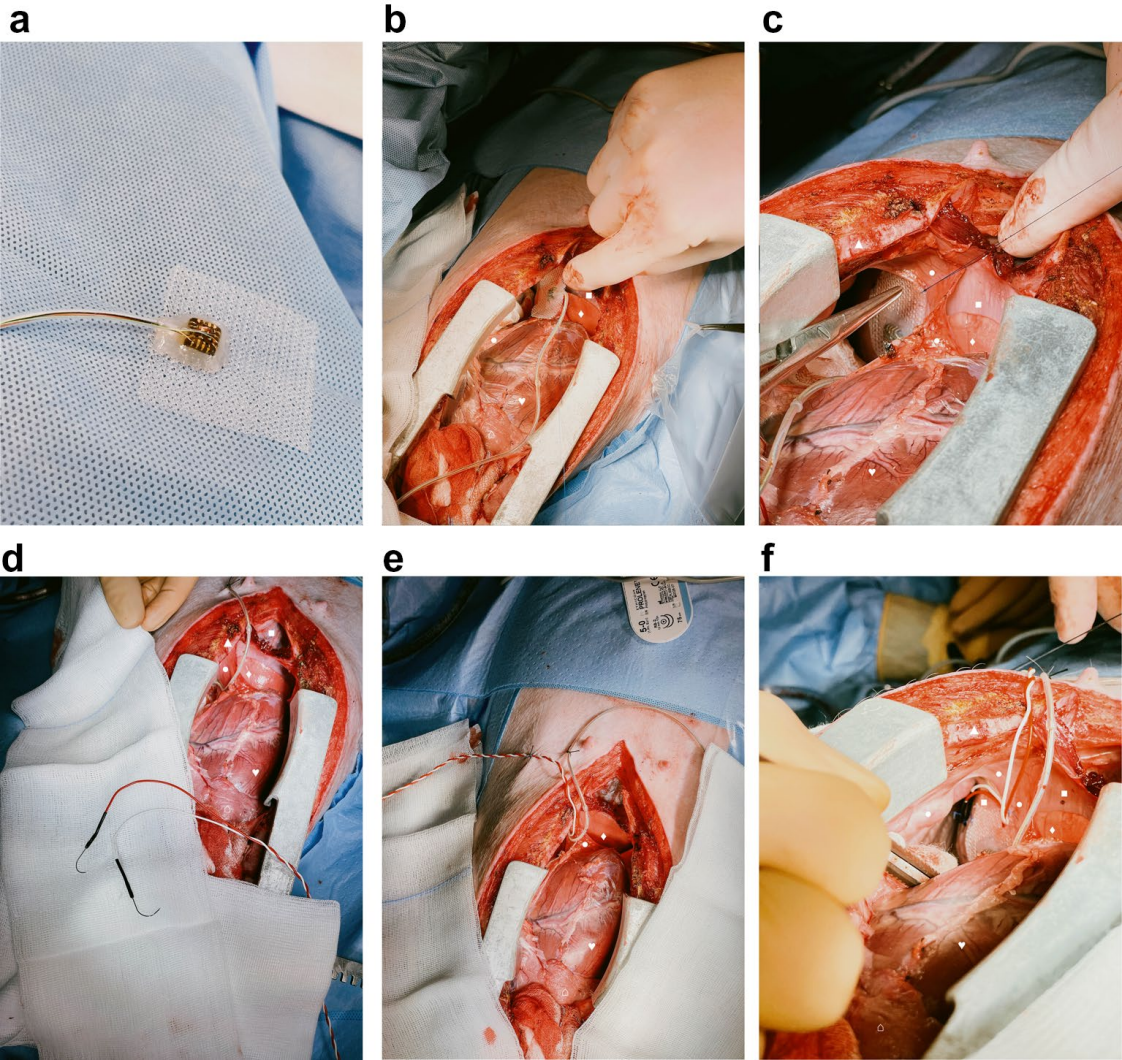
Instrumentation of the left hemi-diaphragm. The orientation is the same in all pictures: the rostral direction is at the bottom and the caudal direction at the top of the picture. ♥ designates the heart.▲ designates the sternum. ● designate the pericardium. ■ designates the diaphragm. ♦ designates the lung. ⌂ designates the thymus. (**a**) Detailed depiction of the accelerometer used in the experiment. (**b**) Positioning of the accelerometer to the most cranial point of the swine’s left hemi-diaphragm. (**c**) Fixation of the accelerometer to the diaphragm via four simple stitches to the edges of the accelerometer. (**d**) Detailed depiction of the hook electrodes used for electromyogram readings. (**e**) Implantation of the hook electrodes to the left hemi-diaphragm. (**f**) Positioning of the hook electrodes as lateral as possible and close to the chest wall with 10 mm distance between its poles.

**Figure 4:**
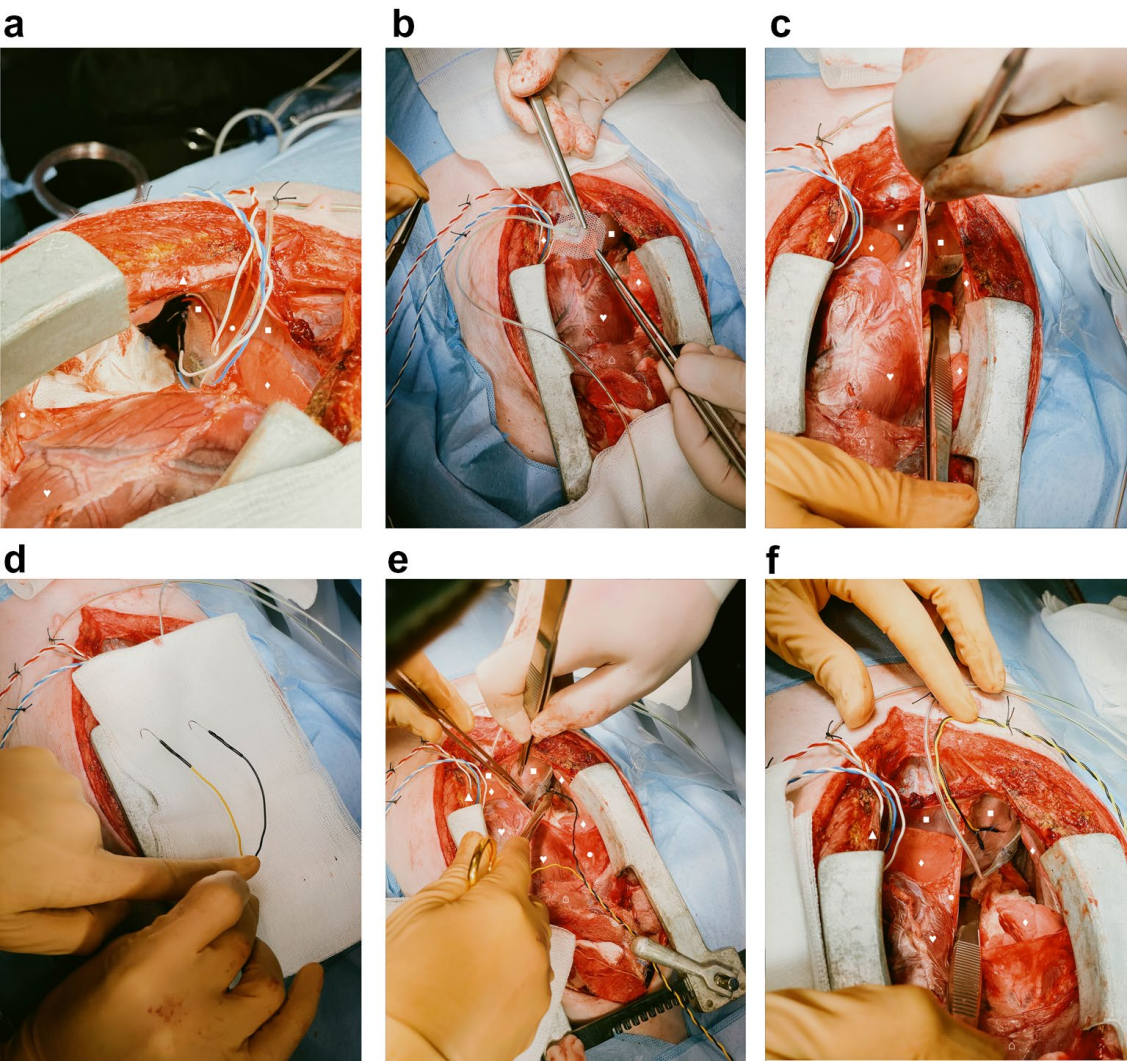
Instrumentation of the right hemi-diaphragm. The orientation is the same in all pictures: the rostral direction is at the bottom and the caudal direction at the top of the picture, except for panel (**a**), in which caudal is top right and rostral bottom left of the picture. ♥ designates the heart.▲ designates the sternum. ● designate the pericardium. ■ designates the diaphragm. ♦ designates the lung. ⌂ designates the thymus. (**a**) Fixation of the cables to the skin directly adjacent to the sternotomy wound with simple stitches. (**b**) Implantation of an accelerometer to the most cranial point of the right hemi-diaphragm. (**c**) Fixation of the accelerometer to the right hemi-diaphragm via simple stitches to the edges of the accelerometer. (**d**) Detailed depiction of the hook electrodes used for diaphragmatic pacing. (**e**) Implantation of the pacing electrodes directly adjacent to the insertion of the phrenic nerve into the diaphragm. (**f**) Fixation of the cables of the implanted devices to the skin. Note the distance of approximately 10 mm between the poles of the pacing electrodes.

Swine were placed in supine position on the operation table (Fig. 1c). Following standard sterile draping, the skin (Fig. 2a) was sharply incised using a type 10 blade. The underlying tissue was cut down using a monopolar knife (Erbe, Tübingen, Germany) until the sternum was exposed (Fig. 2b). It was opened via a median sternotomy using an oscillating bone saw (518.01, Synthes, Bochum, Germany) (Fig. 2c) to achieve exposure of both hemi-diaphragms (Fig. 2d). After horizontal pleural opening and lung displacement by wet gauzes, an accelerometer sensor circuitry (Fraunhofer IBMT, Institute for Biomedical Engineering, Sulzbach, Germany) (Fig. 3a) was positioned at the most cranial point of the left hemi-diaphragm (Fig. 3b). We secured the accelerometer via four individual stitches to its edges using 5-0 polypropylene sutures (Prolene, Ethicon, Nordersted, Germany) [Fig. 3c]. For electromyogram recordings, two hook electrodes (Osypka, Rheinfelden, Germany) [Fig. 3d] were placed 10 mm apart from the accelerometer. The hook electrodes’ position was as close as possible to the lateral chest wall (Fig. 3e), at which we fixed them to the diaphragm using a U-stitch of 5–0 polypropylene (Prolene, Ethicon, Norderstedt) for each electrode (Fig. 3f). After the left hemi-diaphragm was fully instrumented, the lines of the accelerometer and the hook electrodes were affixed to the skin directly lateral to the sternotomy with individual stitches using size 0 polyester fibre sutures (Mersilene, Ethicon, Norderstedt, Germany) [Fig. 4a].

The same steps were repeated on the swine’s right hemi-diaphragm: An accelerometer was also positioned at the most cranial point of the right hemi-diaphragm (Fig. 4b). The accelerometer was affixed to it using again four individual stitches to the edges with 5–0 polypropylene sutures (Fig. 4c). Afterwards, two temporary myocardial pacing electrodes (Osypka, Rheinfelden, Germany) [Fig. 4d] were sutured to the right hemi-diaphragm. The pacing electrodes were localised adjacent to the insertion point of the right phrenic nerve (Fig. 4e), using U-stitches of a polypropylene 6-0 suture with a distance of 10 mm between the pair of electrodes. The hook electrodes were positioned in a similar way as in the left hemi-diaphragm: As lateral as possible and close to the chest wall with a distance of 10 mm between its poles. The electrodes were secured in a similar fashion with U-stitches of a 6-0 polypropylene suture, too. All lines of the pacing electrodes and the accelerometer were secured to the skin, close to the sternotomy wound, by individual stitches of a size 0 polyester fibre suture (Fig. 4f).

**Figure 5:**
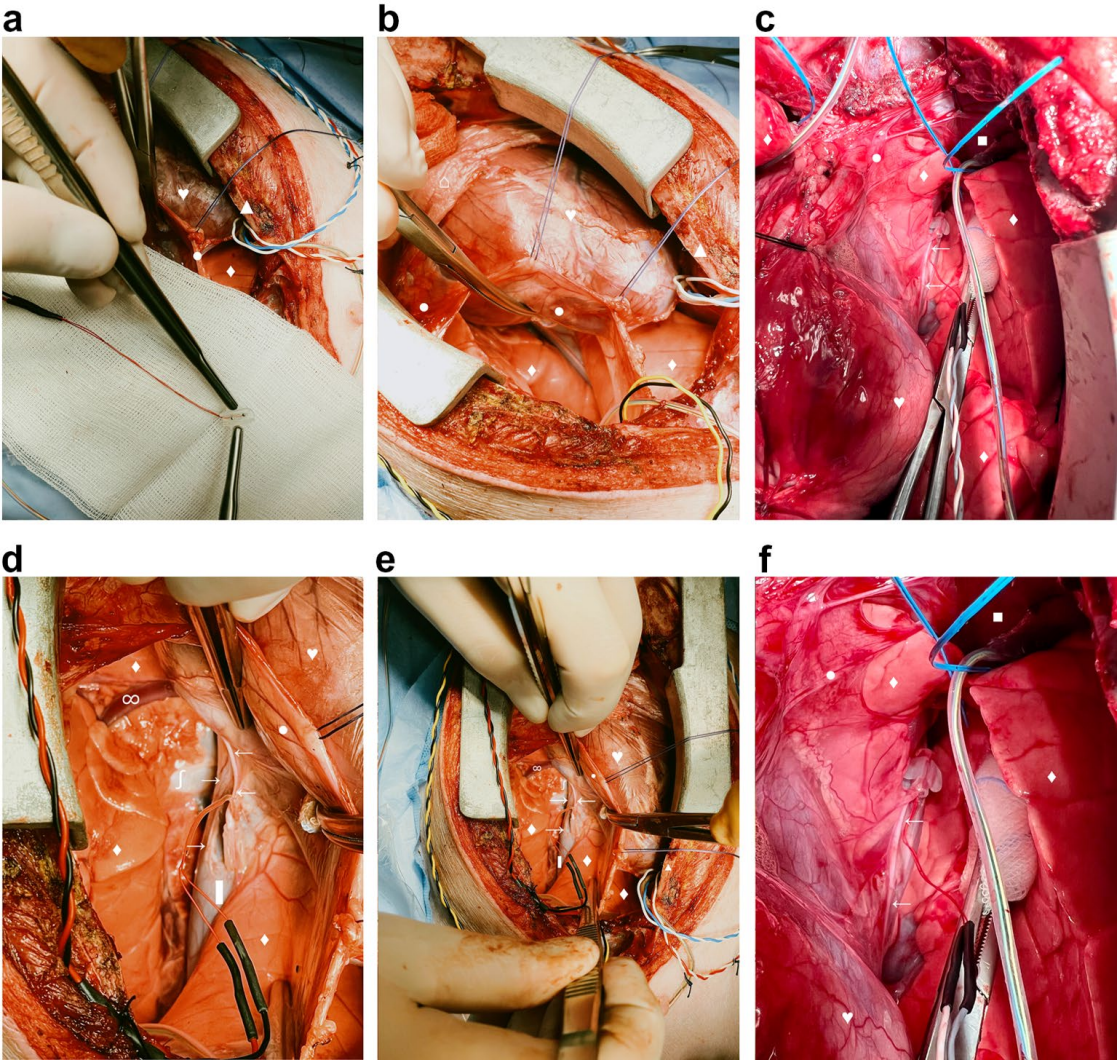
Instrumentation of the right phrenic nerve. The orientation is different to the preceding figures: In panels (**a**) and (**b**), the rostral direction is at the top left and caudal direction is at the bottom right of the picture. In panels (**d**) and (**e**), the rostral direction is at the top and the caudal direction at the bottom of the picture. In panels (**c**) and (**f**), the rostral direction is at the bottom and the caudal direction at the top of the picture. ♥ designates the heart.▲ designates the sternum. ● designate the pericardium. ■ designates the diaphragm. ♦ designates the lung. ← designates the phrenic nerve. → designates the vagus nerve. ▌ designates the oesophagus. ∫ designates the vena cava inferior. ∞ designates the azygos vein. ⌂ designates the thymus. (**a**) Detailed depiction of the silicone-cuff-electrode for bipolar neurostimulation for the phrenic nerve. (**b**) The right pleura was opened and the phrenic nerve identified along its course. (**c**) The intended position of the neurostimulation electrode along the phrenic nerve at the level of the inferior vena cava. (**d**) Increased exposure of the right phrenic nerve after a limited sharp neurolysis to allow placement of the neurostimulator electrode. (**e**) Final setup with all devices implanted. (**f**) Detailed depiction of the neurostimulator electrode at the phrenic nerve.

A silicone-cuff-electrode for bipolar neurostimulation (Fraunhofer IBMT, Institute for Biomedical Engineering, Sulzbach, Germany) (Fig. 5a) was positioned at the right phrenic nerve: Two 3-0 silk stay stitches in the pericardium were used to retract the heart leftward, thereby exposing the space between the medial surface of the right lung and the right circumference of the heart (Fig. 5b). This enabled identification of the right phrenic nerve at the level of the inferior vena cava (Fig. 5c,f**).** A limited neurolysis of around 20 mm was performed by sharp dissection taking care to injure neither the phrenic nerve nor its nutritive vessels (Fig. 5d). After final positioning of the stimulation electrode to the right phrenic nerve (Fig. 5e,f), its lines were secured to the skin with individual stitches of a size 0 polyester fibre suture.

**Figure 6:**
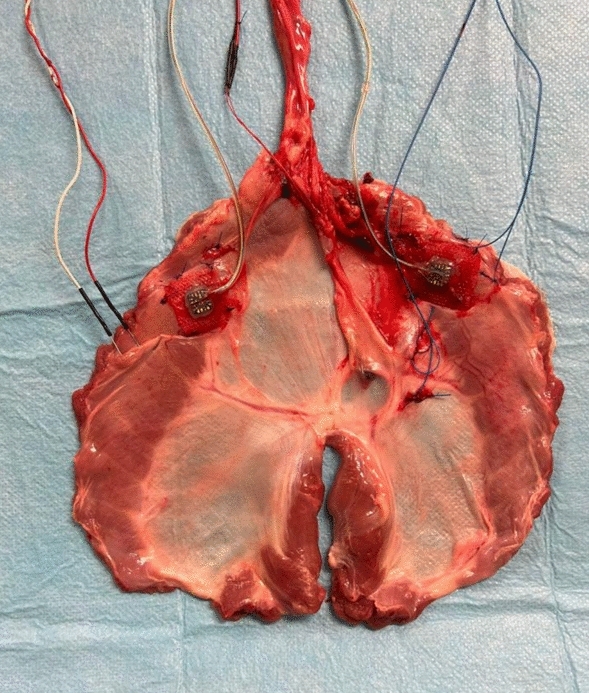
Topview depiction of the experimental setup with all implanted devices affixed to it. The orientation of the specimen corresponds to the viewpoint.

To ensure re-expansion of the lungs after thoracic instrumentation (Fig. 6), end-expiratory pressure was temporarily increased to 10 hectopascal for 15 min. The right hemi-diaphragm could be paced via the stimulation electrodes at the diaphragm or the nerve electrode. This enables identification of different detection thresholds before and after transection of the right phrenic nerve. Right sided diaphragmatic pacing could be triggered by the electromyogram of the intact left hemi-diaphragm. This enables synchronised unilateral diaphragmatic pacing by the non-paretic hemi-diaphragm.

**Figure 7:**
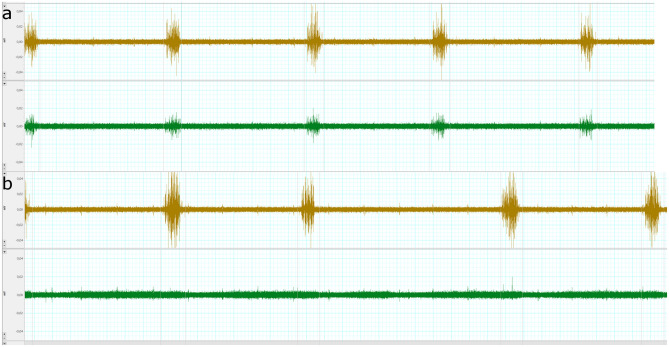
Electromyography readings confirmed unilateral diaphragmatic hemiparesis and success of the external stimulation. The upper curve in both panels represents the left hemi-diaphragm and the lower curve in both panels represents the right hemi-diaphragm. A 100 Hz high pass filter was used. (**a**) The phrenic nerves of both hemi-diaphragms work in parallel and both hemi-diaphragms show muscle activity in parallel as depicted by the electromyography readings. (**b**) Following transection of the right phrenic nerve, the electromyogram of the right hemi-diaphragm does not show any muscle activity, whereas the left hemi-diaphragm shows the same activity as before.

**Figure 8:**
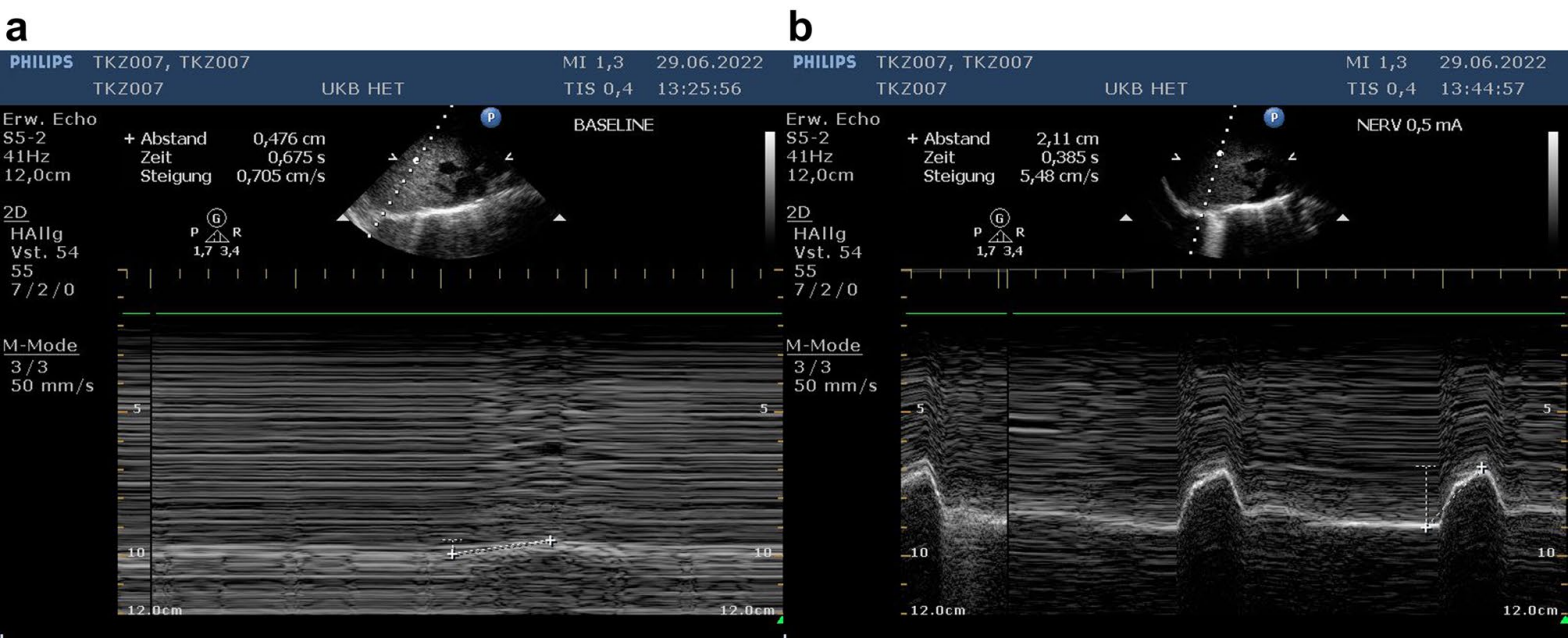
Ultrasound examination of the externally stimulated right phrenic nerve. (**a**) Minimal diaphragmatic excursion without stimulation during the inspiratory cycle of the respirator. (**b**) Regular diaphragmatic excursion following external stimulation of the nerve with a stimulus of 0.5 mA.

Success of the transection of the right phrenic nerve was assessed by the readouts of the electromyogram. Before transection, both hemi-diaphragms did work in parallel (Fig. 7a). Following transection of the right phrenic nerve, muscle activity could only be observed in the left hemi-diaphragm, whereas there was no muscle activity in the right hemi-diaphragm anymore (Fig. 7b). We also visualised the right hemi-diaphragmatic paresis using fluoroscopy, which demonstrated the paresis under spontaneous respiration (Supplemental video). Successful stimulation could be demonstrated using ultrasound as a different method of assessment: During the inspiratory cycle of the respirator, there was minimal passive movement of the right hemi-diaphragm (Fig. 8a). Following external stimulation at the right phrenic nerve, there was substantial movement of the right hemi-diaphragm indicating successful external stimulation (Fig. 8b).

**Figure 9:**
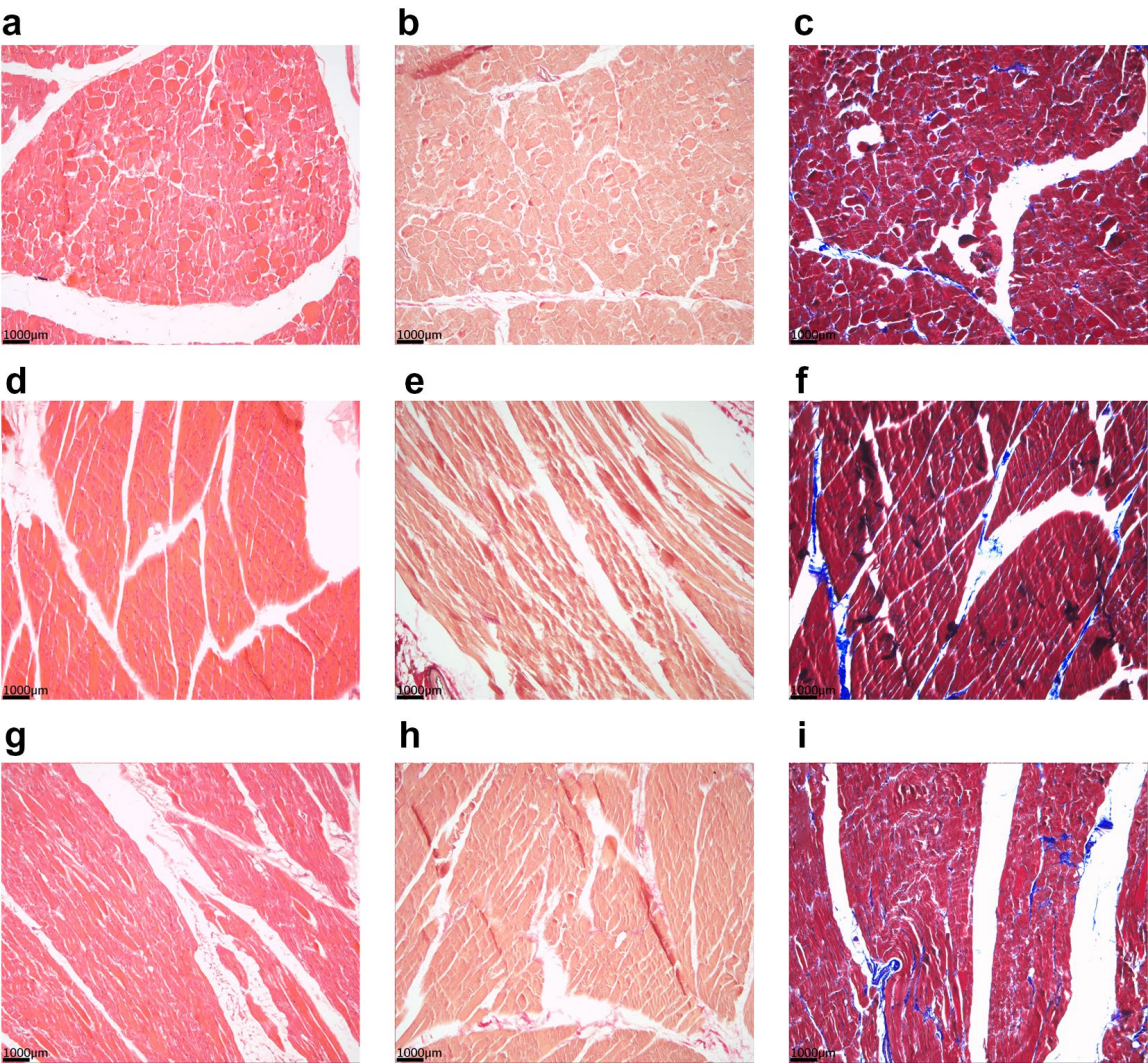
Histologic analyses revealed no damage to the diaphragm. All panels represent a 10-times magnification. Panels (**a**–**c**) represent a cross-sectional view of the paced diaphragm. Panels (**d**–**f**) represent a longitudinal view of the paced diaphragm. Panels (**g**–**i)** represent the cross-sectional view of a healthy diaphragm and shows no difference compared to the paced diaphragm. In panels (**a**), (**d**), (**g**) Hematoxylin–eosin staining was used, in panels (**b**), (**e**), (**h**) Elastica-van Gieson staining was used, and in panels (**c**), (**f**), (**i**) Masson’s trichrome stain was used.

The pigs were euthanized after the experiments were finished. Histologic changes of the diaphragm due to direct pacing were not observed in cross-sectional (Fig. 9a–c) or longitudinal views (Fig. 9d–f), as comparison to an untouched diaphragm revealed (Fig. 9g–i).

## Discussion

Swine have been the model of choice in investigating the effects of phrenic nerve palsy since the 1980s, but with varying intensity^13–17^. Recently, research interest on this topic was gaining ground again: A model of unilateral phrenic nerve injury by thoracoscopic ligation has been developed in Landrace pigs^18^. In addition, the respiratory physiology of hemi-diaphragmatic paresis has been evaluated in mixed-breed Norwegian-Yorkshire pigs^19^. This supports the notion that the pig is an accepted model for pre-clinical research in diaphragmatic dysfunction. Although there is no perfect animal model to mirror a human condition^20^, one may strive to achieve meaningful results by coming as close as possible to human anatomy and physiology^21^. Swine have physiologic characteristics similar to humans, particularly in the first days of life^22^, which made them the favourite animal model of childhood diseases^23^. It has been shown that porcine thoracic and abdominal organ weights can be similar to those of infants^20^. This also supports the use of swine to model diaphragmatic dysfunction following heart surgery.

Among several other factors, word count limits have been identified to negatively impact reproducibility, because word count limits require research to be condensed in the shortest possible form^24^. A systematic review assessed compliance with the ARRIVE-guidelines in reports of pig models: They found that husbandry conditions, experimental outcomes, and statistical analyses were often poorly reported^25^. Detailed reporting is crucial not only for replication, but also for other laboratories aiming to use a previously described model. Usage of the same model by other groups would result in higher reproducibility due to increased heterogeneity^26^. Consequently, model descriptions were gaining ground in specialist^27–29^ and generalist^30,31^ journals in order to enhance reproducibility of experimental animal studies. To contribute to this aim, we opted to separate model description and construction from dissemination of results. In the present form, we could avoid to fell prey to word limits or maximum numbers of figures that would result in the omission of necessary details. The illustrated step-by-step description allows replication of our animal model by other groups.

Compared to existing models of hemi-diaphragmatic paresis^18^, our model offers the advantage to be modified to model all steps of a single-ventricle circulation. The thoracic organs were already exposed by the sternotomy. This potential expansion is crucial, because these children are at particular risk for phrenic nerve palsy due to recurring operations^32,33^. Moreover, children with single ventricles are more severely affected by hemi-diaphragmatic palsy, as it results in a poorly performing Fontan circulation^34^, which may subsequently lead to a failing Fontan circulation^35^. This emphasises the importance of a well-documented, expandable, and reproducible model of hemi-diaphragmatic paresis to evaluate direly needed potential new treatments. Due to its step-by-step description with accompanying detailed photographic depiction of all steps, our report allows reproduction of the model generation by other groups. Thereby, the model can be used for additional experiments by others and hopefully contribute to further improvement in the care of these severely affected children in the long run.

## Methods

We used five German landrace swine, four males and one female, each being an experimental unit, weighing x̄̄ = 19.9 (95% confidence interval 16.85–22.95) kg. Five animals were used because Monte-Carlo simulations demonstrated that five animals should be sufficient for a pilot study^36^. Swine were supplied in-house by the Agricultural school, Rheinische Friedrich–Wilhelms–University Bonn, Bonn, Germany. Pigs were of conventional microbiologic status and had an acclimatisation period of three days at our facility. The temperature was regulated between 16 and 18 °C with a relative humidity between 50 and 60%. The air within the facility was exchanged at least 8 times per hour. Swine were housed alone within their box of 4 to 6 square meters that was enriched with chains, balls, and additional material to play with (Fig. 1a). An infrared heating lamp was available to the pig anytime to ensure active warming in the resting periods. We used dark–light cycles of twelve hours with artificial lighting between 7 and 19 o’clock. Drinking water was supplied ad libitum and the pigs were fed regular chow (Altromin 9023, Altromin Spezialfutter, Lage, Germany).

Swine had undergone a preoperative fasting period of twelve hours. Experiments started in the morning and only one swine was operated per fortnight to clearly separate the experiments into independent mini-experiments^37^. Premedication consisted of intramuscularly applied ketamine (20 mg/kg) [WDT, Garbsen, Germany] in combination with azaperone (2 mg/kg) [Richter Pharma, Wels, Austria] and atropine (0.02 mg/kg) [B.Braun, Melsungen, Germany]. After adequate sedation was achieved, venous access was implemented with a 1.1 mm outer diameter Jelco® catheter (Smith Medical, Grasbrunn, Germany) in one of the ear veins. Anaesthesia induction consisted of piritramide (0.5 mg/kg) [Hameln Pharma, Hameln, Germany] and propofol (10 mg/kg) [CP Pharma, Burgdorf, Germany]. Muscle relaxation was avoided to decrease the risk of malignant hyperthermia. We secured the airway via endotracheal intubation using a straight size 4 Miller blade with a 5.0 mm internal diameter curved, microcuffed endotracheal tube (Avanos, Hamburg, Germany). For invasive ventilation, we used a Servo-i (Maquet, Rastatt, Germany) with synchronised intermittent mandatory ventilation with a frequency around 15 per minute, an inspiratory pressure of 15 cmH_2_O and a positive end-expiratory pressure of 5 cmH_2_O. General anaesthesia was maintained with continuous intravenous infusion of propofol (1–5 mg/kg) via a Perfusor®Space (B.Braun, Melsungen, Germany) combined with repeated doses of piritramide (0.2–0.5 mg/kg) and supplemented by occasional single doses of ketamine (5–10 mg/kg) or midazolam (0.5 mg/kg) [B.Braun, Melsungen, Germany]. We monitored the depth of the total intravenous anaesthesia with the Narcotrend-system via a CompactM-monitor for intraoperative use (Narcotrend, Hannover, Germany). Needle electrodes (Neuroline Twisted Pair Subdermal, 12 × 0.4 mm, Ambu, Ballerup, Denmark) were placed at the standard positions according to the manufacturer’s instructions. During the procedure, swine had the electroencephalographic Kugler-stadium^38^ D0. This stage corresponds to general anaesthesia.

The swines’ vital signs were monitored using an Infinitiy C500 monitor (Dräger, Lübeck, Germany) for electrocardiogram, pulse oximetry and invasive blood pressure. For capnometry, we used Datex-Ohmeda S/5 (Datex-Ohmeda, Duisburg, Germany) (Fig. 1b). We inserted an arterial line (2.7 French leadercath, Vygon, Aachen, Germany) in the right femoral artery for continuous blood pressure monitoring and a three-lumen central line (5.5 French, Teleflex, Fellbach, Germany) in the right femoral vein (Fig. 1c). Arterial blood gas analyses were analysed in a RapidPoint® 500 machine (Siemens Healthineers, Erlangen, Germany). We also placed a 10 Charrière transurethral catheter with an included thermistor (Asid Bonz, Herrenberg, Germany) to monitor urine output and intravesical temperature. Temperature was also measured with a 9 French oesophageal probe (Smiths Medical, Grasbrunn, Germany). Maintenance fluids were infused at a rate of x̄ = 316.49 mL/h (95% confidence interval: 158.59–474.39) using Ionosteril 1/1 (Fresenius Kabi, Bad Homburg, Germany).

Diaphragmatic excursion was assessed using ultrasound in a subxiphoid plane with M-mode using a Philips HD 15 (Philips, Amsterdam, The Netherlands) and a S5–2.5 MHz sector array transducer.

At the end of the experiment, swine were euthanized using T61® (tetracaine/mebezonium/embutramide) [Intervet, München, Germany] at a dose of 0.5 mL/kg. The diaphragm was explanted and 1 × 1 × 0.3 cm tissue pieces from the diaphragm were stretched on cork plates, immersed in 4% (pH 6.9) buffered formaldehyde solution (Sigma Aldrich, Darmstadt, Germany) and fixed for 12–24 h at room temperature. We cut a longitudinal and a cross section of each sample, dehydrated the specimens in ascending alcohol concentrations and embedded them in Tissue-Tek III paraffin wax (Sakura Finetek, Alphen aan de Rhijn, Netherlands). Sections of 3 µm thickness were cut, stained with hematoxylin–eosin and Elastica-van-Gieson (Medite Tissue Stainer, Medite, Burgdorf, Germany), according to our routine protocols. In addition, Masson’s trichrome staining was performed for each specimen following the manufacturer’s protocol (Avantor, VWR, Darmstadt, Germany). Pictures of slides were captured using the pathoZoom camera system (Smart in Media AG, Cologne, Germany) attached to a Leica DM 2500 microscope (Leica, Wetzlar, Germany).

Experiments were conducted in accordance with the German Animal Welfare Act and its subsequent statutory acts, which are in accordance with the Council of Europe Convention ETS 123. The competent state agency—*Landesamt für Natur, Umwelt und Verbraucherschutz Nordrhein-Westfalen*—approved the study (permit: 81-02.04.2020.A392). Our study complied with the ARRIVE-guidelines 2.0^39^.

### Supplementary Information

Supplementary Video 1.

## Data Availability

All data generated or analysed during this study are included in this published article.
